# Editorial: Data-intensive medicine and healthcare: ethical and social implications in the era of artificial intelligence and automated decision-making

**DOI:** 10.3389/fgene.2023.1280344

**Published:** 2023-09-07

**Authors:** Aviad Raz, Jusaku Minari, Silke Schicktanz, Tamar Sharon, Gabriele Werner-Felmayer

**Affiliations:** ^1^ Department of Sociology and Anthropology, Ben-Gurion University of the Negev, Beer-Sheba, Israel; ^2^ Uehiro Research Division for iPS Cell Ethics, Center for iPS Cell Research and Application (CiRA), Kyoto University, Kyoto, Japan; ^3^ Department of Medical Ethics and History of Medicine, University Medical Center Göttingen, Göttingen, Germany; ^4^ Department of Ethics and Political Philosophy and Interdisciplinary Hub on Digitalization and Society, Radboud University, Nijmegen, Netherlands; ^5^ Institute of Biological Chemistry, and Bioethics Network Ethucation, Medical University Innsbruck, Innsbruck, Austria

**Keywords:** genomic medicine, ethics, personalized medicine, automated decision-making, artificial intelligence

Medical “big data” and artificial intelligence (AI) are a hyped duo. Promises include developing more personalised treatments, delegating medical decision-making to tireless and seemingly objective algorithms, improving preventive screening, and providing healthcare more efficiently through predictive risk scores. AI and big data, however, do not automatically transform into improved health outcomes. The practical and functional uses of AI in big data environments require integrating and interpreting a wide variety of medical data (e.g., from genomics or other omics, imaging, biomarker analyses) and other personal data. As a result, AI-driven technology bears various new challenges and risks at the societal, algorithmic, organizational, expert, and individual levels.

Scholarship on the ethical, legal, and social issues of using AI in data-intensive medicine and healthcare has highlighted numerous areas of contention, including regulation, explainability, privacy, data sharing and protection, trust, and biases, as well as how AI might affect the patient–doctor relationship and support interdisciplinary expert teams in their decisions. Aiming to extend this perspective, this Research Topic focuses on AI applications in various areas of innovative data-intensive medicine, such as genomics, neuroscience, and child and elderly care. The contributions explore how ethical and social considerations can/should be part of medical AI by considering issues of diversity, the significance of datafication and automation, public and patient participation, developing deliberative or open science approaches (such as open codes), and how to ensure interoperability among developers and users while preventing misuse, hacking, or manipulation. The Research Topic comprises 10 articles dealing with various aspects of the prospects and perils of AI in healthcare, which can be grouped into several themes representing key concerns in this emerging field—especially regulation, data sharing, and explainability.

Rubeis et al. can be read as a prolegomenon to the Research Topic, as they introduce a useful typology of the various ways in which “democratizing AI” is used to hype the field of AI in healthcare. Their study highlights the ways in which the concept of “democratizing AI” tends to frame patients as consumers and focus on free-market solutions, while omitting the deliberative processes and modes of participation needed to ensure that those affected by AI in healthcare have a say on its development and use. These needs and lacunae are further highlighted in the other articles.

The required and/or missing regulation and ethical embedding of AI-assisted healthcare are discussed in four articles. Stake and Heinrichs examine the ethical aspects of e-health applications for child health screening. They propose to develop age-specific models that consider the vulnerability of children to balance their right to informational self-determination with medical needs. Meszaros et al. examine more generally the future directions of AI regulation in medical care implied by the proposed EU AI Act and the EU General Data Protection Regulation, analysing ways to harmonize the principles of data protection and ethical AI. Fritzsche et al. discuss the recent use of AI for polygenic risk scores (PRSs), which may enable higher prediction accuracy but also presents a range of increasingly complex ethical challenges regarding fairness, trust, and explainability, as well as regulatory uncertainties. The authors strongly advocate a proactive approach to embedding ethics in research and implementation processes for AI-driven PRSs. Raz and Minari expand this discussion by comparing AI-derived ethnicity-related PRSs and social scoring, both of which, while representing different applications, may reproduce biases. The authors argue that if AI-derived PRSs evaluate or classify the risks of natural persons based on their ethnic/racial self-designations, this will be akin to AI-derived social scoring based on previous social behaviours in multiple contexts or known or predicted personal or personality characteristics.

The challenges of data sharing are explored in two articles. Reer et al. review the requirements for useful data sharing in human neuroscience. They discuss international legal frameworks and the standardization of data and metadata organization and annotation. Bak et al. criticize the conventionally used “either/or” choice of the “consent or anonymize approach” and its challenge to balancing data privacy and data access. They argue that the “AI revolution” in healthcare can be realized only through transnational data sharing governance policies.

Two articles address the issue of explainability. Pierce et al. discuss the opacity problem of AI in clinical use by drawing a distinction between the function of explainability for the current patient and that for the future patient. They argue that in day-to-day clinical practice, accuracy is sufficient as an “epistemic warrant” for clinical decision-making and that the most compelling reason for requiring explainability in the sense of scientific or causal explanation is its potential to improve future care. Ott and Dabrock suggest that while transparency often follows an “all or nothing” logic, intelligibility offers the opportunity to uncover the essential elements of an AI system: Does the system provide an adequate basis for rendering people intelligible? And does it do so not only ex ante during data collection and algorithm design but continuously during implementation and adaptation and, finally, ex post after the actual use case?

Finally, Schicktanz et al. suggest a novel approach not only to embedding ethics into the development and use of medical AI (as all the articles discuss for their respective fields) but also to integrating AI into the development of ethical assessment. They argue for AI-assisted ethical simulation that can improve context-sensitive ethical analyses, as well as for thought experiments and future-oriented technology assessments—for example, applications catering for persons with dementia or cognitive impairment.

The diversity of the articles included in this Research Topic reminds us that under no circumstances should groups exclusively pursuing their own interest dominate the debate on medical AI. Rather, addressing the ethical challenges of medical AI requires interdisciplinary efforts involving computer scientists, ethicists, sociologists, policymakers, and domain experts (such as healthcare professionals) to address the multiple aspects of this debate which should be open to all the stakeholders involved.

